# Checklists to improve the quality of the orthopaedic literature

**DOI:** 10.4103/0019-5413.40251

**Published:** 2008

**Authors:** Raman Mundi, Harman Chaudhry, Ishu Singh, Mohit Bhandari

**Affiliations:** Department of Surgery, McMaster University, Hamilton, Ontario, Canada; 1 Division of Orthopedic Surgery, Millcreek Community Hospital, Erie, PA, USA

**Keywords:** Critical appraisal, checklists, meta-analysis, diagnostic tests, quality assessment, randomized trials

## Abstract

Several checklists have been developed in an effort to help journals and researchers improve the quality of reporting in research. The CONSORT statement and the CLEAR NPT evaluate randomized trials. The MOOSE and QUOROM checklists evaluate meta-analyses. The STROBE checklists assists readers in evaluating observational studies and the STARD checklist was developed for diagnostic test evaluation. The checklists presented here provide an invaluable source of guidance to authors, journal editors and readers who are seeking to prepare and evaluate reports. As evidence-based medicine continues to establish itself as the new paradigm by which medicine is practiced, the need for good reporting for all research designs must also become commonplace as opposed to the exception.

## INTRODUCTION

The current quality of reporting in both the medical and surgical literature is poor and in need of immediate improvement.^1^–^5^ The orthopaedic literature is no exception. In fact, many studies have highlighted the substandard quality of reporting in even ‘high quality’ study designs such as randomized controlled trials and systematic reviews.^6^–^9^

The true benefits of well conducted studies with valid results can only be realized if they are presented to readers in a comprehensive and transparent manner. Owing to the need for improved reporting, several checklists have been developed to guide authors preparing manuscripts for various types of study designs. Although some journals have endorsed certain checklists with marginal improvements in reporting, the quality is still often poor as authors do not adhere to many of the checklist recommendations.^5^,^10^

There is a dire need to promote awareness and understanding of these available checklists so that authors can begin taking advantage of these invaluable guides. The objective of this report is to introduce several of the existing checklists for various study designs. In particular, the study designs focused on in this article include randomized controlled trials, systematic reviews, observational studies, diagnostic trials and qualitative studies.

## CHECKLISTS FOR RANDOMIZED CONTROLLED TRIALS

Although randomized controlled trials (RCTs) are considered the ‘gold standard’ study designs for evaluating treatment effectiveness, they nevertheless remain subject to bias unless methodological and statistical safeguards are implemented into the trial. Briefly, such safeguards include allocation concealment, blinding, ensuring complete patient follow-up and analyses according to the intention-to-treat principle. This is just a sample of many safeguards and other components of randomized trials that should be reported in trial manuscripts. For instance, there are methodological issues unique to nonpharmacological trials (NPTs) that also merit reporting, such as standardization of the intervention and ensuring adequate care provider skill.

To aid investigators in preparing comprehensive and high quality manuscripts for randomized trials, two checklists have gained much attention: 1) the Consolidated Standards of Reporting Trials (CONSORT) statement and 2) the Checklist to Evaluate a Report of a Nonpharmacological Trial (CLEAR NPT).

**Figure 1:**
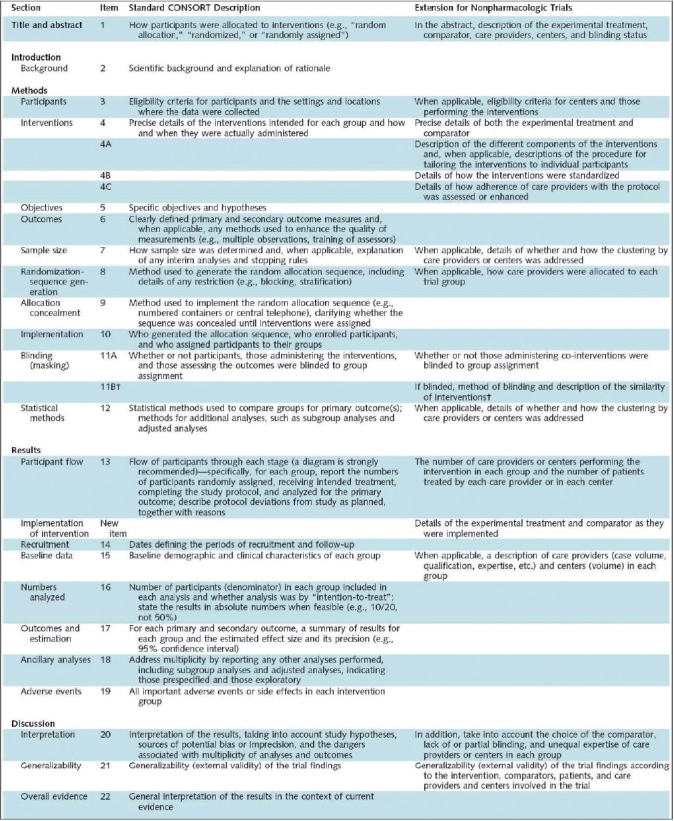
CONSORT checklist^12^

**Figure 2:**
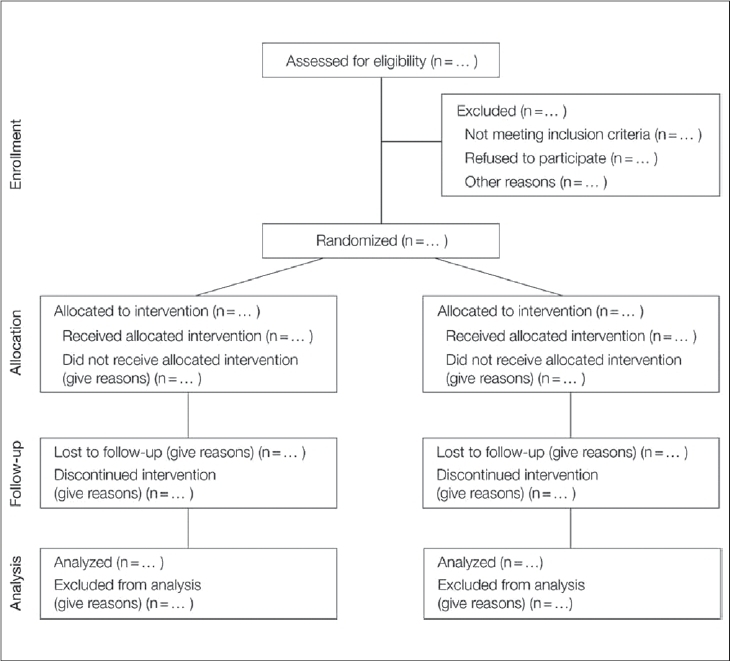
Standard CONSORT flow diagram^11^

**Figure 3:**
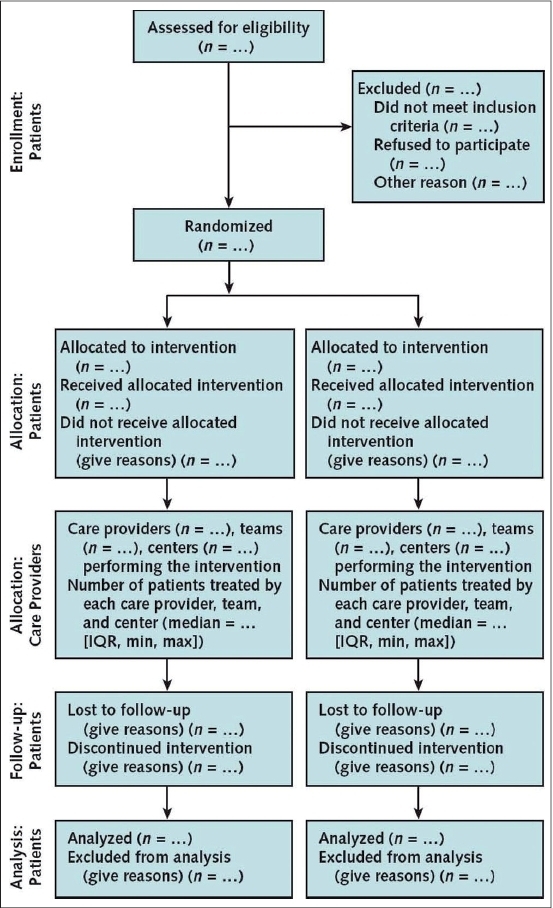
CONSORT flow diagram for Nonpharmacological Trials^12^

The CONSORT statement was first published in The Journal of the American Medical Association in 1996 and revised in 2001. This statement consists of a 22-item checklist and flow diagram that serve as a detailed set of recommendations on how to prepare a report for a randomized trial or conversely, aid in critically appraising the reports of others. In particular, the checklist provides recommendations on how to report the design, analysis and interpretation of the study, whereas the flow diagram offers guidance on how to report the progress of participants through the trial.^11^ Realizing that randomized trials of nonpharmacological interventions have unique challenges—such as complex interventions and difficulty blinding patients—not fully addressed by the revised CONSORT statement, an extension to the CONSORT was recently made to specifically address the issues facing these trials.^12^ Both the standard CONSORT checklist and the modified version for NPTs can be seen in Figure 1. Figure 2 illustrates the standard CONSORT flow diagram and Figure 3 illustrates the modified flow diagram for NPTs. Adherence in the orthopaedic literature to the CONSORT recommendations has been demonstrated to be poor.^8^,^9^ For instance, Bhandari and colleagues^8^ evaluated the reports of 196 randomized trials investigating fracture care across 32 journals and found that the average report adhered to only 32% ± 29% of the CONSORT criteria.

**Figure 4:**
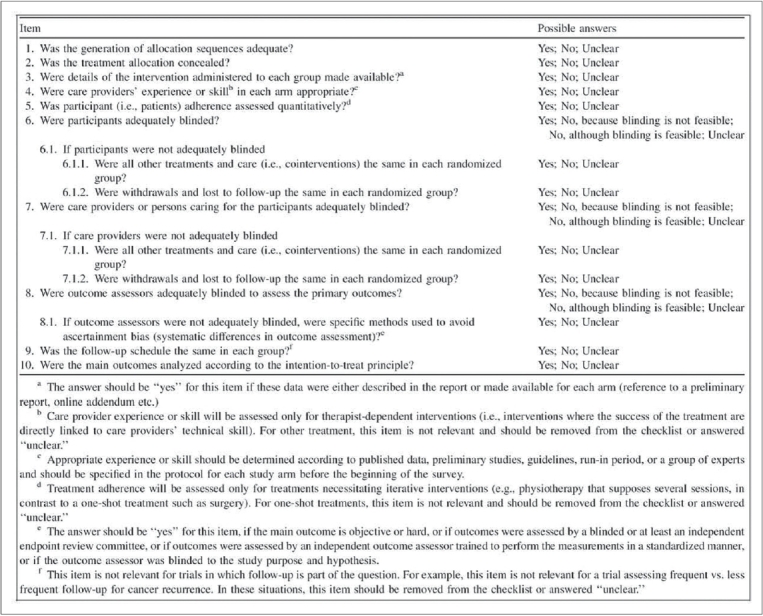
CLEAR NPT checklist^13^

Developed in 2005, the CLEAR NPT is a 15-item checklist (10 main items and 5 sub-items), that serves to critically appraise the reports of randomized trials of nonpharmacological interventions [Figure 4].^13^ As implied by its name, this checklist is useful for assessing nonpharmacological trials due to its focus on key methodological issues surrounding NPTs. Each item on the checklist can be answered with a quick Yes, No or Unclear, making it an efficient tool to evaluate the literature with. Further supporting the claims of studies utilizing the CONSORT statement, Chan and Bhandari^14^ have demonstrated that the quality of reporting for randomized trials in the orthopaedic literature as assessed by the CLEAR NPT is also suboptimal. Although this checklist's primary utility is to evaluate reports, it still serves as a useful guide to authors preparing manuscripts of randomized trials.

## CHECKLISTS FOR SYSTEMATIC REVIEWS AND META-ANALYSES

With the plethora of studies being published constantly, summarizing the results of primary articles on a given topic is a useful and helpful practice for health care providers and policy makers.^15^ Systematic reviews are considered high quality evidence due to their systematic approach at collecting, critically appraising and synthesizing data from original articles on a specific topic. If a quantitative analysis is performed to arrive at a single best estimate of the treatment effect, these reviews are better known as meta-analyses. Due to their systematic nature and ability to put forth a single best estimate of the treatment effect, meta-analyses can have a significant impact on patient care. However, meta-analyses may vary in their methodological rigor and produce results of varying credibility. For instance, the quality of a systematic review or meta-analyses is directly dependent on the quality of the studies included. Thus, systematic reviews and meta-analyses that consider only RCTs would provide stronger evidence than those which consider non-randomized studies as well. However, between 1996 and 2001, it was found that the majority of orthopaedic systematic reviews published in peer-reviewed journals outside the Cochrane collaboration included non-randomized trials.^16^ Clinicians reading the reports of systematic reviews and meta-analyses must be able to appraise the methods and validity of the study in order to confidently interpret their results.

**Figure 5:**
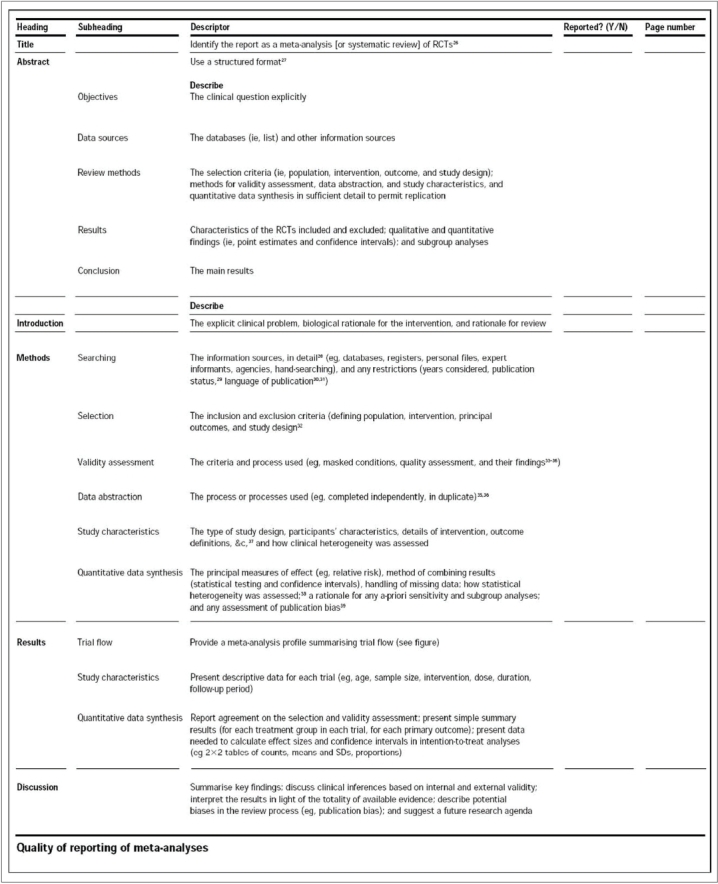
QUOROM checklist^15^

**Figure 6:**
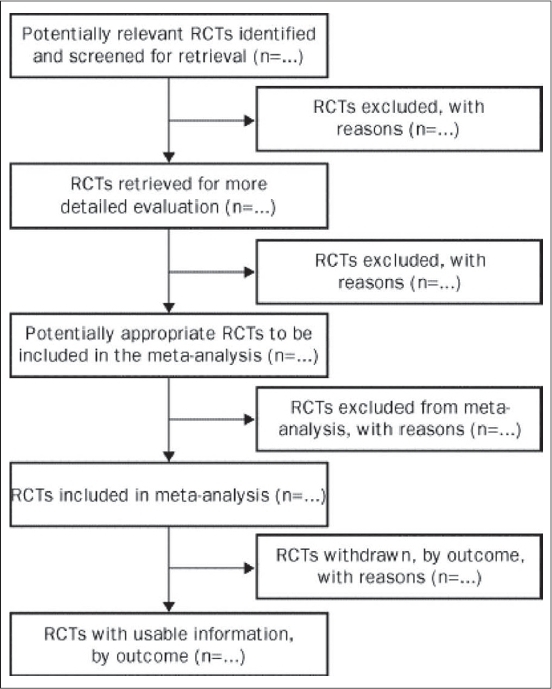
QUOROM flow diagram^15^

The Quality of Reporting of Meta-Analyses (QUOROM) statement, consisting of an 18-item checklist and flow diagram, was developed to aid authors preparing reports of meta-analyses of RCTs [Figure 5 and 6].^15^ The QUOROM checklist outlines a set of recommendations on how to prepare the abstract, introduction, methods, results and discussion sections of a meta-analysis. The ultimate goal of these reporting guidelines is to provide readers with transparency regarding the search, selection, validity assessments, data abstraction, study characteristics, quantitative data synthesis and trial flow of the study.^15^ For instance, under the “methods” section of the checklist, authors are encouraged to report the criteria used to assess the quality of the included RCTs and the outcome of such quality assessments. This is imperative, as RCTs with deficiencies in certain methodological safeguards have been shown to produce biased results.^5^ Incorporating these ‘biased’ studies without caution into a meta-analysis would also result in a biased estimate of the treatment effect in the meta-analysis. The purpose of the flow diagram is to help authors on reporting details of the inclusion and exclusion of RCTs. Although the QUOROM statement is designed specifically to guide reporting of meta-analyses, authors of systematic reviews of RCTs can also benefit from these recommendations—with the exception of the reporting recommendations geared towards the quantitative analysis, as this step is only carried out in a meta-analysis.^15^

Not all meta-analyses can rely solely on RCTs to answer a question of interest. First and foremost, RCTs are relatively scarce in the orthopaedic literature, making it impractical to always exclusively use data from RCTs.^6^ Secondly, for issues surrounding risk factors for disease and harm, it would be unethical to randomize patients to groups in which they would be subject to any potentially harmful risks.^17^ For instance, if the question of interest was, “What is the effect of smoking on fracture nonunion rates?”, it would not be feasible to carry out a randomized study in which patients were randomly allocated to either a smoking or non-smoking group.^18^ As a result, several meta-analyses rely upon observational studies - those in which patients are naturally exposed to risk factors or in which physician or patient preference determines allocation to a treatment or control intervention.^19^ The importance of comprehensive reporting for such meta-analyses can not be overstated, as observational studies are more prone to biased results than RCTs. In fact, arriving at a single estimate of the effect of a treatment or exposure when pooling data from observational studies requires extreme caution, as these results can often be misleading.^16^

**Figure 7:**
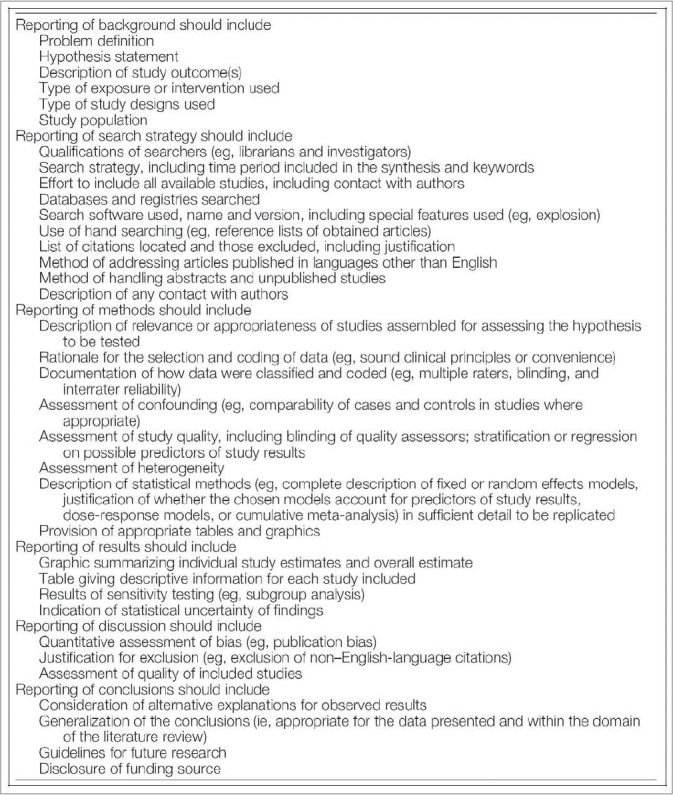
MOOSE checklist^17^

In 2000, the Meta-Analyses of Observational Studies in Epidemiology (MOOSE) group produced a 35-item checklist that details how meta-analyses of observational studies should be reported. Specifically, the checklist provides recommendations on how to report background information, the search strategy and the methods, results, discussion and conclusion sections of the paper [Figure 7]. For each of these six categories, there are several corresponding items that are listed by the MOOSE groups as essential for reporting. For instance, under search strategy, reports should include the qualifications of the searchers, a detailed description of the search strategy and the method by which articles in foreign languages were utilized, among other details regarding the search.^17^

## CHECKLISTS FOR OBSERVATIONAL STUDIES

As mentioned, not all research questions can be answered through RCTs. Therefore, observational studies have an important role in answering questions of treatment effectiveness and disease etiology. Three primary observational study designs include the cohort, case-control and cross-sectional studies. Briefly, cohort studies usually follow two groups of patients; one group in which everyone has been exposed to a risk factor or treatment and the other in which no exposure has occurred. The groups are then compared for the rate of development of disease or outcome of interest. In case-control studies, a group that has already developed an outcome of interest is compared to a group without the outcome for factors that may be associated with the outcome. Cross-sectional surveys are carried out at a single time point, at which both the outcome and factors of interest are measured. Due to the lack of randomization, observational studies are inherently more prone to potential biases. Even if investigators attempt to match groups for known prognostic factors, there may be underlying imbalances in unknown prognostic factors that may produce misleading and biased results.^20^ Furthermore, case-control studies are always retrospective in nature (cohort studies may also be retrospective) which increases the potential for incomplete and biased data collection.^18^ Despite these limitations, observational studies have a crucial role to play in medical research and as such, satisfactory reporting to allow readers to evaluate these studies is of utmost importance.

**Figure 8:**
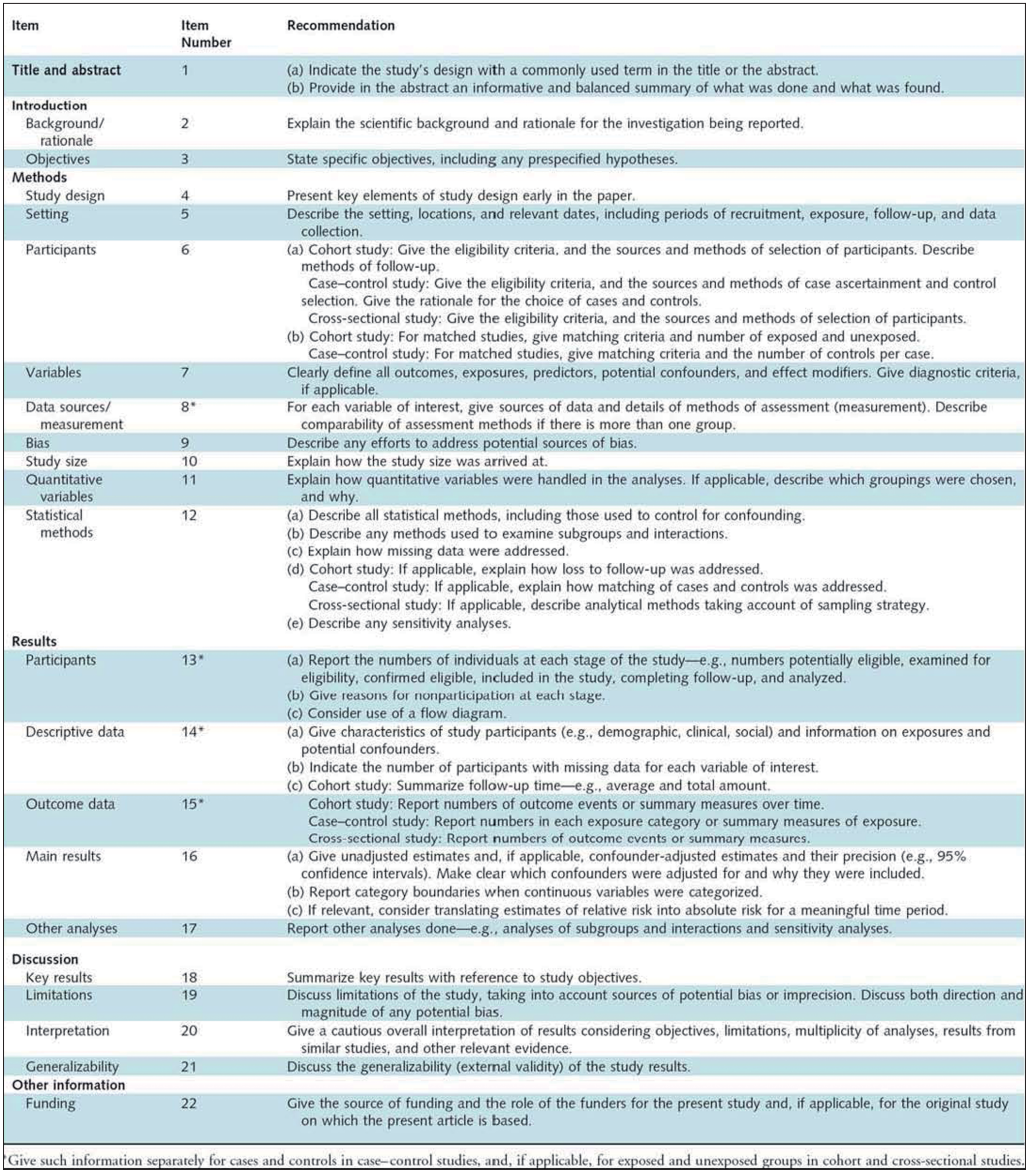
STROBE checklist^22^

The Strengthening the Reporting of Observational Research in Epidemiology (STROBE) statement, outlines how to prepare good manuscripts for these three observational study designs. It consists of a 22-item checklist which provides reporting recommendations for all sections of the paper, as well as on funding sources [Figure 8]. Of the 22-items, 18 are general to all three study designs and 4 are design-specific. In particular, information in the methods section regarding participants (item 6) and statistical methods (item12), as well as in the results section regarding descriptive data (item 14) and outcome data (item 15) are design-specific. Although the STROBE group emphasizes that reporting of all 22-items in this checklist is essential, they encourage authors to utilize their preferences and creativity when selecting the order and format of presenting such details.^21^

## CHECKLISTS FOR STUDIES OF DIAGNOSTIC ACCURACY

Diagnostic tests are widely used by clinicians to diagnose health states and subsequently initiate, alter or terminate various treatment options.^22^ Diagnostic studies evaluate the accuracy of a diagnostic test (by its level of agreement to the current ‘gold standard’ for diagnosis) in predicting a disease, stage of a disease, health status or any health condition that could prompt clinical action.^22^ The ‘gold standard’ is typically impractical to use in regular clinical encounters and therefore the study is attempting to offer a more practical alternative.^23^ As such, studies of diagnostic test accuracy have the potential to directly impact treatment decisions and, therefore, patient care. Unfortunately, it has been demonstrated that methodologically compromised studies are more likely than methodologically rigorous studies to overestimate the accuracy of diagnostic tests.^24^ In the hands of the uncritical clinician, such poor studies may lead to the unwarranted use and interpretation of a diagnostic test, ultimately to the detriment of high quality patient care.

**Figure 9:**
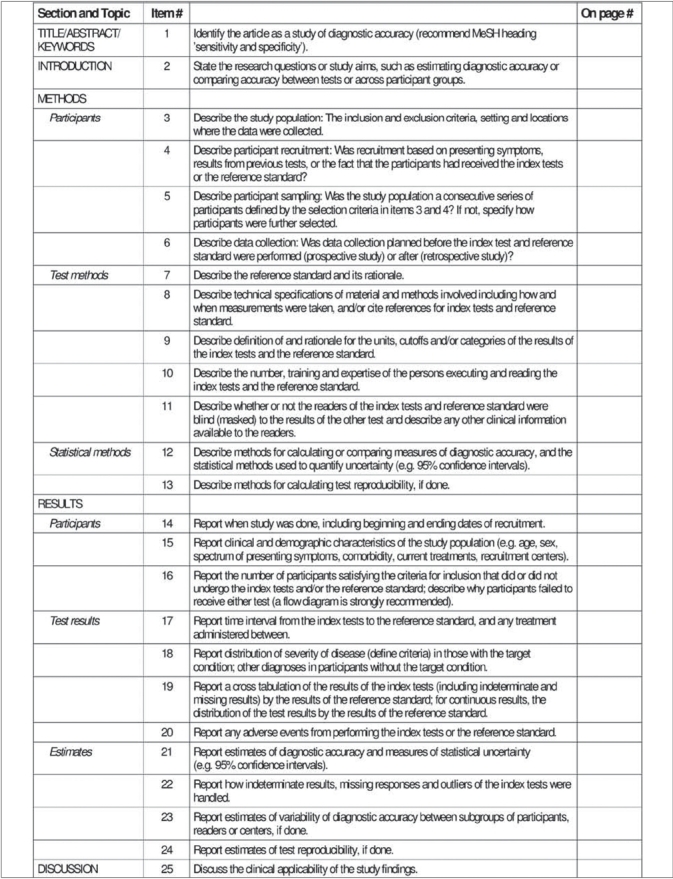
STARD checklist^27^

**Figure 10:**
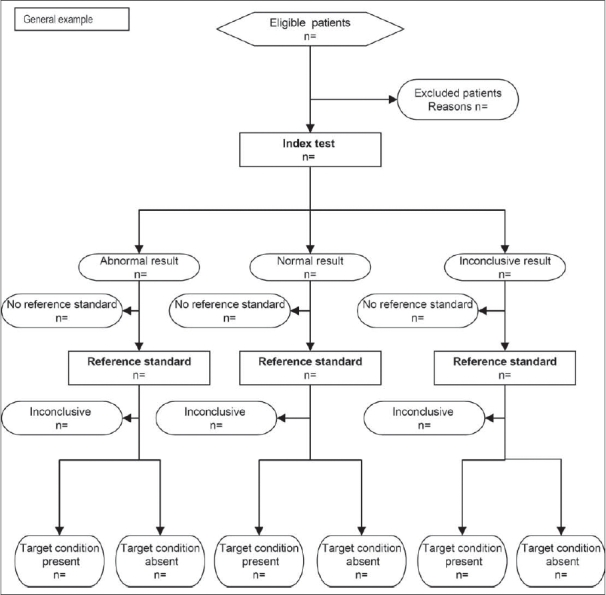
STARD flow diagram^27^

Recognizing the importance of studies evaluating diagnostic accuracy, Bossuyt and colleagues^25^ developed the Standards for the Reporting of Diagnostic Accuracy studies (STARD) statement. The STARD statement includes a 25-item checklist which outlines crucial information that authors should include in the abstract, introduction, methods, results and discussion sections of a report to enable an adequate assessment of both external validity (i.e. how generalizable study results are) and internal validity (i.e. the potential for bias) [Figure 9].^26^ In addition to some relatively common elements, such as inclusion/exclusion criteria, method of data collection and method of data analysis, the STARD checklist also includes some unique items. For instance, it asks for a description and rationale of the gold standard to which the diagnostic test (referred to as the index test) is being compared. This is because even positive study results will be limited by the effectiveness of the ‘gold standard’ as a diagnostic tool.^23^ The STARD statement also includes and encourages authors to use a flow diagram to report the number of patients included and excluded in the diagnostic and/or ‘gold standard’ tests [Figure 10].^26^

Rama and colleagues recently published an investigation of diagnostic accuracy studies using the STARD criteria in three orthopaedic journals.^27^ They found that the majority of studies had deficiencies in reporting of methodology and, overall, reported less than two-thirds of the STARD criteria. Currently, no major orthopaedic journals have adopted the STARD statement;^28^ this may be partly attributable to the scarcity of diagnostic accuracy studies, which constitute only 1% of the orthopaedic literature.^27^ However, owing to the enormous implications that a newly implemented diagnostic test can have on patient care, we believe that the STARD statement must be endorsed to enable readers to adequately interpret study results and prevent untenable treatment decisions.

## CHECKLISTS FOR QUALITATIVE STUDIES

Qualitative studies are useful in the surgical literature for comprehensively describing phenomena (from social, emotional and experiential perspectives) as well as for generating hypotheses that can subsequently be quantitatively verified or disproven.^29^,^30^ Giacomini, Cook and Guyatt have stated that qualitative studies can provide a “rigorous alternative to armchair hypothesizing”.^29^ For instance, one study published in the Journal of Bone and Joint Surgery (American Volume) explored the reasons why (from the patient's perspective) many elderly arthritic patients are unwilling to undergo a total joint replacement procedure.^31^ The principles of evidence-based medicine demand that articles should be critically appraised before results are implemented into clinical practice. However, the methodological rigour of qualitative studies has come under criticism; commentators have stated that there is a need for rigorous methodological standards in order to minimize the effect of bias on study results.^32^,^33^

**Figure 11:**
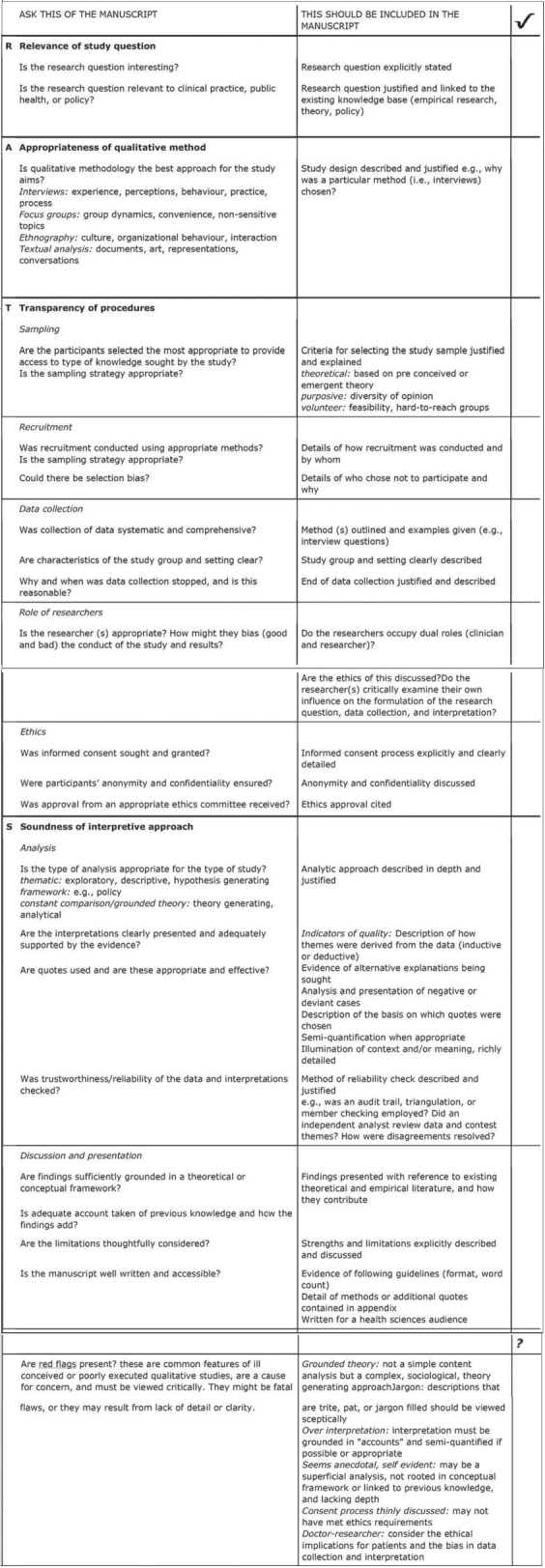
RATS checklist^35^

The most comprehensive available checklist for qualitative studies is the RATS guidelines developed by Clark and adopted as a 28-item checklist by BioMed Central in the instructions to authors section [Figure 11].^34^,^35^ RATS is an acronym which describes four components of a rigorously reported qualitative study: 1) Relevance of the study question; 2) Appropriateness of qualitative method; 3) Transparency of procedures; and 4) Soundness of interpretive approach.^35^ In addition to the 28-item checklist, the RATS guideline offers a section on possible “red flags” authors should avoid.^34^ Unfortunately, there does not appear to be an overwhelming consensus on the effectiveness of this particular checklist to ensure all pertinent methodological criteria have been met, as there is for the CONSORT criteria. However, authors of qualitative studies are advised to consider and incorporate the checklist criteria in reporting findings.

## CONCLUSION

For over a decade now, several checklists have been developed in an effort to help investigators prepare reports for a variety of different study designs. If the time, effort and resources put forth in carrying out medical research is to make its impact on patient care and policy decisions, then the importance of complete and comprehensive reporting can not be overstated. The checklists presented here provide an invaluable source of guidance to authors, journal editors and readers who are seeking to prepare and evaluate reports. To gain greater information on these and other available reporting checklists, we encourage readers to locate the original articles in which these checklists are published. Furthermore, certain checklists, such as the CONSORT statement, have corresponding explanation and elaboration papers which are informative and aid in promoting understanding of the checklists. As evidence-based medicine continues to establish itself as the new paradigm by which medicine is practiced, the need for good reporting for all research designs must also become commonplace as opposed to the exception.
